# Case report: Rubella virus-induced cutaneous granulomas in a girl with atypical SCID caused by *DCLRE1C* gene mutations

**DOI:** 10.3389/fgene.2023.1115027

**Published:** 2023-03-16

**Authors:** Sihan Deng, Shijia Rao, Alun R. Wang, Wei Shi

**Affiliations:** ^1^ Department of Dermatology, Xiangya Hospital, Central South University, Changsha, Hunan, China; ^2^ Department of Dermatology, The Second Xiangya Hospital, Central South University, Hunan Key Laboratory of Medical Epigenomics, Changsha, Hunan, China; ^3^ Department of Pathology, Tulane University School of Medicine, New Orleans, LA, United States; ^4^ National Clinical Research Center for Geriatric Disorders, Xiangya Hospital, Central South University, Changsha, Hunan, China

**Keywords:** next-generation sequencing, rubella virus, whole-exome sequencing, *DCLRE1C* gene, granulomatous, severe combined immunodeficiency syndrome

## Abstract

Here, we report a case of rubella virus-induced granulomatous dermatitis in a young girl with immunodeficiency caused by *DCLRE1C* gene mutations. The patient was a 6-year-old girl who presented with multiple erythematous plaques on the face and limbs. Biopsies of the lesions revealed tuberculoid necrotizing granulomas. No pathogens could be identified on extensive special stains, tissue cultures, or PCR-based microbiology assays. Metagenomic next-generation sequencing analysis revealed the rubella virus. Underlying atypical severe combined immunodeficiency was recognized based on the patient’s history of repetitive infections since birth, low T-cell, B-cell, and NK cell counts, and abnormal immunoglobulins and complements. Whole-exome sequencing revealed the genetic abnormality of the atypical severe combined immunodeficiency (SCID), and compound heterozygous mutations of the *DCLRE1C* gene were detected. This report highlights the diagnostic values of metagenomic next-generation sequencing in identifying rare pathogens causing cutaneous granulomas in patients with atypical SCID.

## 1 Introduction

Cutaneous granulomatous dermatitis is a common disorder that can be divided into infectious and non-infectious etiologies. Infection should be ruled out if no clear cause is identified, especially in immunocompromised patients. Even if an infectious etiology is suspected, the causal microorganisms are often unidentifiable. The prevalence of granulomas is rather high among patients with combined immune deficiency and other types of immunodeficiencies. Skin represents a primary site of granulomas in patients with combined immune deficiency. Some granulomas are caused by the rubella virus infection. The traditional ways of identifying microorganisms are based on morphology, histochemistry, and cultures with limited sensitivity and efficiency. Targeted PCR assays developed in recent years have improved sensitivity significantly. Metagenomic next-generation sequencing (mNGS) is an unbiased assay that identifies a broad spectrum of pathogens, including many rare pathogens. Mutations in the *DCLRE1C* gene have been documented to cause immunodeficiency with phenotypes ranging from severe combined immunodeficiency to mere antibody deficiency. The whole-exome sequencing technique is an effective tool in revealing underlying molecular mechanisms causing immunodeficiencies.

## 2 Manuscript

### 2.1 Case description

**FIGURE 1:**
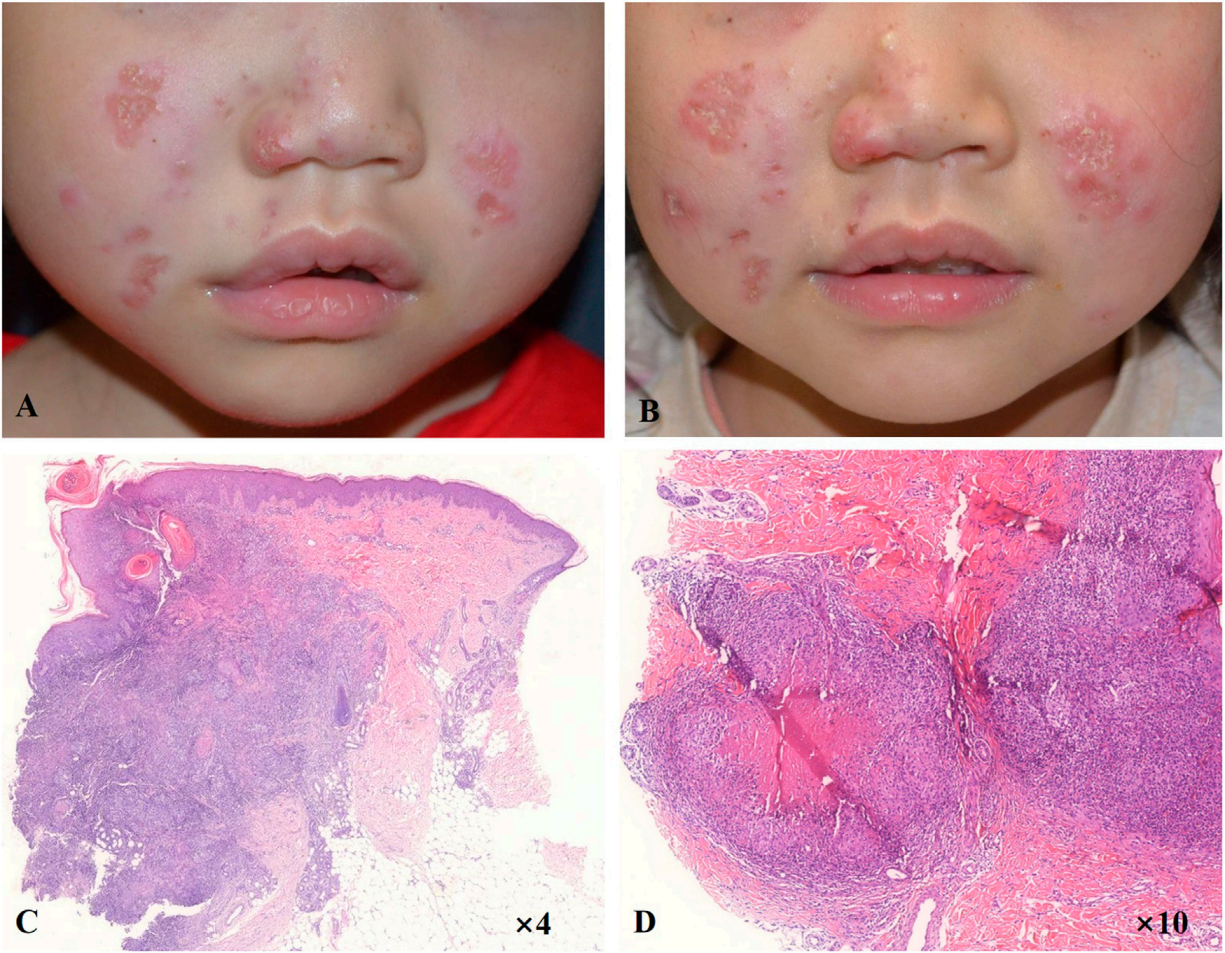
RuV infection-induced cutaneous granulomatous dermatitis. Erythematous plaques on cheeks at visit **(A)** and after 10-month follow-up **(B)**. **(C)** Dense lymphohistiocytic infiltrate in the dermis forming a nodule. **(D)** Necrotizing granulomas in the deep dermis.

**FIGURE 2:**
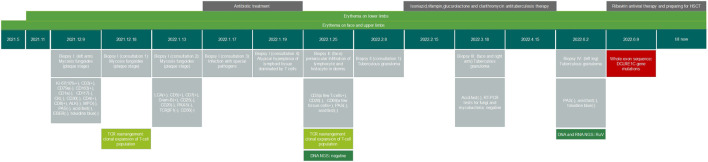
Timeline of the patient’s care.

A 6-year-old girl presented with facial erythematous plaques that had gradually spread into her limbs over the course of a year (Figure 1A). The center of some erythematous plaques was ulcerated and scabbed spontaneously. She appeared to be predisposed to respiratory infections since birth. Multiple biopsies of the plaques revealed necrotizing granulomatous inflammation (Figure 1D). No microorganisms were identified on PAS and acid-fast stains, tissue cultures, and PCR assays for fungi and mycobacteria. Dense lymphocyte infiltrate with lymphoid follicle formation in the dermis with adnexal involvement was also found in a biopsy from the left upper arm (Figure 1C). Most lymphocytes were T-cells (CD3^+^) with mixed CD4^+^ and CD8^+^ cells. Some lymphocytes showed mild cytological atypia. Clonal expansion of the T-cell population was detected through T-cell receptor gene rearrangement studies. The patient had neutropenia and lymphopenia of T cells, B cells, and NK cells. Specifically, cell counts of CD3^+^ T cells, CD4^+^ T cells, and CD8^+^ T cells were all lower than normal. Subpopulations of the CD3+/CD8-/IL-4+ lymphocytes and CD3+/CD8-/IFN-γ+ lymphocytes were higher than normal, and CD3+/CD4+/CD25+/FOXP3+ lymphocytes and CD3+/CD8-/IL-17A + lymphocytes were lower than normal. Serum IgA and IgM levels were significantly lower, and the patient’s C3 level was slightly lower than normal (Table 1). No clinically significant nodular metabolic enhancement was observed on PET-CT. The timeline of the patient’s care is summarized in Figure 2.

**TABLE 1:** Partial laboratory results of this patient.

	Result	Normal range
CD3^+^ lymphocytes (%)	**34.93**	50–84
CD3^+^CD4^+^ lymphocytes (%)	51.11	30.00–67.00
CD3^+^CD8^+^ lymphocytes (%)	41.19	23.00–50.00
CD3^+^CD8^−^ lymphocytes (%)	58.81	50.00–75.00
CD3^+^CD8-IL-4+ lymphocytes (%)	**3.86**	0.30–2.20
CD3^+^CD8-IFN-γ lymphocytes+ (%)	**38.06**	6.50–28.00
CD3^+^CD4^+^CD25+FOXP3+ lymphocytes (%)	**3.25**	4.10–9.40
CD3^+^CD8-IL-17A + lymphocytes (%)	0.65	0.20–2.40
CD4^+^ cell (/uL)	**258**	550–1,440
CD8^+^ cell (/uL)	**185**	320–1,250
B lymphocyte (%)	**3.62**	5–18
B lymphocyte (/uL)	**23**	90–560
NK cell (/uL)	**128**	150–1,100
Total T lymphocyte (/uL)	**486**	955–2,860
C3 (g/L)	**0.6**	0.7–1.4
C4 (g/L)	0.215	0.1–0.4
IgG (g/L)	5.59	5.4–13.4
IgM (g/L)	**0.11**	0.43–1.52
IgA (g/L)	**0.12**	0.3–1.48
IgE (IU/mL)	1	0–100
Blood routine		
WBC (×10^9/L)	**2.52**	3.5–9.5
Neutrophils (×10^9/L)	**1.44**	1.8–6.3
Lymphocytes (×10^9/L)	**0.49**	1.1–3.2
Monocytes (%)	**12.7**	3–10

In bold: values above or below the reference range.

Because mycobacterial infections were not ruled out, a diagnostic therapeutic 8-week course of quadruple antituberculosis therapy was performed but without effect. Repeated metagenomic DNA and RNA mNGS analyses were performed, and rubella virus (RuV, read 7280) was detected. A serology test showed RuV IgM was 1.41 S/CO (positive range:≥1), and the RuV IgG titer was 243.7 IU/mL (positive range:≥10). Therefore, a diagnosis of rubella virus-induced cutaneous granulomatous dermatitis was reached.

**FIGURE 3:**
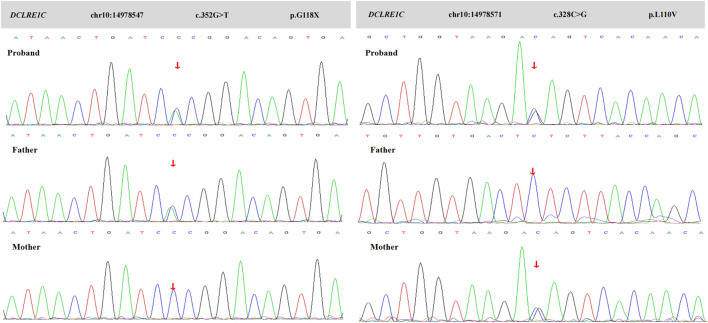
Sanger sequence of this family. Chromatograms show that her father is heterozygous for c.352G>T (p.G118X), and her mother is heterozygous for c.328C>G (p.L110V). The proband shows a compound heterozygote of these mutations.

Considering RuV-associated granulomas are rare and almost exclusively occur in the context of immune deficiency, and the patient had signs of immunodeficiency, a whole- exome sequencing was performed, and heterozygous mutations in *DCLRE1C* gene as c.352G>T (p.G118X) and c.328C>G (p.L110V) were detected (Figure 3). It was concluded that hypomorphic heterozygous mutations of *DCLRE1C* genes led to an atypical SCID in this girl, who developed RuV-induced cutaneous granulomatous dermatitis, likely after inoculation with the rubella virus through vaccination. The patient had been waiting for hematopoietic stem-cell transplantation (HSCT) for the previous 10 months, and no other treatments had been initiated. An increase in the number and size of plaques on her face and limbs had been noted (Figure 1B), and the plaques had remained asymptomatic. The patient was hospitalized for pre-transplant examination and preparation.

### 2.2 Discussion

Here, we report the first Chinese patient with RuV-induced cutaneous tuberculoid necrotizing granulomas in an immunodeficient child with heterozygous *DCLRE1C* gene mutations. Our patient received an MMR vaccine injection when she was 18 months old. The lesions were likely induced by vaccine-derived live attenuated rubella viruses. In addition, a clonal expansion of the T-cell population in the granulomatous lesions was detected. No obvious evidence of a cutaneous T-cell lymphoma was identified. However, it has been observed that immunodeficiency could lead to a clonal expansion of lymphocyte populations, potentially as an autoreactive proliferation of T cells in response to infections or autoantigens, as likely happened in this patient (Herber et al., 2020).

In 2014, vaccine-derived rubella virus was identified from cutaneous granulomas in patients with primary immunodeficiency (Bodemer et al., 2014). Since then, no more than 100 cases have been reported worldwide, mostly in European and American populations. Pathologically, RuV infections could present as necrotizing granulomas, although they can also present as non-specific dermal chronic inflammation (Buchbinder et al., 2019). Skin lesions have been documented to develop several years on average after measles–mumps–rubella (MMR) vaccination, for which attenuated rubella virus is administered. Wild-type RuV has also been detected much less often in granulomatous lesions (Shields et al., 2021).

Based on the reported cases, all RuV-associated granuloma patients present with some degree of immune deficiency, although some are clinically immunocompetent adults (Wanat et al., 2022). Many patients have DNA repair defects, with *DCLRE1C* gene mutations as one of the causes. *DCLRE1C* encodes ARTEMIS, which is essential in the V(D)J recombination of the immunoglobulin and T-cell receptor genes in T- and B-cell development, as well as in DNA repair (Felgentreff et al., 2015). Mutations in the *DCLRE1C* gene can lead to severe combined immunodeficiency, Omenn syndrome, and radiosensitivity (Strubbe et al., 2021). Severe combined immunodeficiency is an inherited, most severe form of primary immunodeficiency caused by mutations in genes involved in lymphocyte development and function and characterized by the absence or dysfunction of T lymphocytes. It affects both cellular and humoral adaptive immunity. B lymphocytes and NK cells may be affected as well. Over 20 different molecular defects are documented. These include defects in genes involved in antigen receptor gene rearrangement, T-cell receptor signaling, T-cell differentiation, thymic development, and thymic egress of T cells.

Patients with hypomorphic mutations, however, may present with less severe clinical phenotypes depending on residual activity levels on the alleles, causing so-called leaky or atypical SCID (Felgentreff et al., 2015). Atypical SCID occurs and may be defined as a primary immunodeficiency disease secondary to hypomorphic mutations in SCID-causing genes with a milder presentation and higher T-cell counts than typical SCID. The T-cell count is generally above 100/μL (Felgentreff et al., 2011). In atypical SCID, patients usually survive beyond 12 months of age. Atypical SCID has been diagnosed in all age groups, frequently significantly later than SCID.

Our patient had a long history of repetitive respiratory tract infections and recurrent nasal stuffiness since birth. These infections are generally not severe and could be alleviated after a few days on antibiotics. A high copy number of Epstein–Barr virus (EBV) DNA was detected in her serum, which would be an indicator that the patient was experiencing an active EBV infection. The patient presented with reduced numbers of peripheral T cells, B cells, NK cells, and neutrophils, and low IgM and IgA levels. Despite these abnormalities, she seemed relatively normal in development and overall health. Her conditions, therefore, fit into atypical SCID.

The pathogenesis of overall white blood cells and immunoglobulin abnormalities caused by *DCLRE1C* gene mutations remains unknown. Usually, the *DCLRE1C* gene variants cause T^−^/B^−^/NK^+^ SCID. However, our patient presents with a unique T^−^/B^−^/NK^−^ phenotype. She presents with a novel compound heterozygosity of two mutations, c.352G>T and c.328C>G. Mutation of c.352G>T (p.G118X) is a nonsense mutation that would very likely result in gene dysfunction. No mutation of this site has been reported in the database, and the ClinVar database assesses it as pathogenic. The clinical significance of mutation of c.328C>G (p.L110V) is uncertain according to ACMG guidelines. Mutations at this location have been reported, although clinical phenotypes (Shahbazi et al., 2019; Xiao et al., 2021). The presence of compound mutations could make a clear correlation between particular genotypes and phenotypes difficult or impossible.

There are no effective conventional therapies for RuV-associated granulomatous diseases. The efficacy of antiviral therapy, TNF-α, IL-1R antagonists, and glucocorticoids remains uncertain (Perelygina et al., 2021). HSCT is considered a curative treatment, and the treatment is more likely to succeed when it is performed early in the disease process. The caveat is that patients with Artemis-deficient immunodeficiency have a decreased tolerance to the alkylating agents used as preparative regimens for HSCT and that carry a risk of significant long-term toxicity (Schuetz et al., 2014). Therefore, it is important to explain the benefits and risks of HSCT in detail to the patients or their parents. Hematopoietic stem cell transplantation is underway for our patient.

Metagenomic next-generation sequencing is a powerful new platform that can simultaneously identify genetic material from entirely different kingdoms of organisms. It can sequence all nucleic acids in a sample and identify multiple populations of microorganisms from different taxa and their proportions. The potential clinical applications are tremendous, particularly in the diagnosis of infectious diseases. As a less biased, sensitive, and broad-spectrum assay, it opens doors for revealing pathogens in numerous unsolvable cases per traditional methodologies.

## Data Availability

The data sets presented in this study can be found in online repositories. The names of the repository/repositories and accession number(s) can be found at: https://www.ncbi.nlm.nih.gov/, PRJNA905930.

## References

[B1] Bodemer C. Sauvage V. Mahlaoui N. Cheval J. Couderc T. Leclerc-Mercier S. (2014). Live rubella virus vaccine long-term persistence as an antigenic trigger of cutaneous granulomas in patients with primary immunodeficiency. Clin. Microbiol. Infect. 20 (10), O656–O663. 10.1111/1469-0691.12573 24476349

[B2] Buchbinder D. Hauck F. Albert M. H. Rack A. Bakhtiar S. Shcherbina A. (2019). Rubella virus-associated cutaneous granulomatous disease: A unique complication in immune-deficient patients, not limited to DNA repair disorders. J. Clin. Immunol. 39 (1), 81–89. 10.1007/s10875-018-0581-0 30607663 PMC7739844

[B3] Felgentreff K. Lee Y. N. Frugoni F. Du L. van der Burg M. Giliani S. (2015). Functional analysis of naturally occurring DCLRE1C mutations and correlation with the clinical phenotype of ARTEMIS deficiency. J. Allergy Clin. Immunol. 136 (1), 140–150. 10.1016/j.jaci.2015.03.005 25917813 PMC4494888

[B4] Felgentreff K. Perez-Becker R. Speckmann C. Schwarz K. Kalwak K. Markelj G. (2011). Clinical and immunological manifestations of patients with atypical severe combined immunodeficiency. Clin. Immunol. 141 (1), 73–82. 10.1016/j.clim.2011.05.007 21664875

[B5] Herber M. Mertz P. Dieudonné Y. Guffroy B. Jung S. Gies V. (2020). Primary immunodeficiencies and lymphoma: A systematic review of literature. Leuk. Lymphoma 61 (2), 274–284. 10.1080/10428194.2019.1672056 31580160

[B6] Perelygina L. Faisthalab R. Abernathy E. Chen M. H. Hao L. Bercovitch L. (2021). Rubella virus infected macrophages and neutrophils define patterns of granulomatous inflammation in inborn and acquired errors of immunity. Front. Immunol. 12, 796065. 10.3389/fimmu.2021.796065 35003119 PMC8728873

[B7] Schuetz C. Neven B. Dvorak C. C. Leroy S. Ege M. J. Pannicke U. (2014). SCID patients with ARTEMIS vs RAG deficiencies following HCT: Increased risk of late toxicity in ARTEMIS-deficient SCID. Blood 123 (2), 281–289. 10.1182/blood-2013-01-476432 24144642 PMC3953035

[B8] Shahbazi Z. Yazdani R. Shahkarami S. Shahbazi S. Hamid M. Sadeghi-Shabestari M. (2019). Genetic mutations and immunological features of severe combined immunodeficiency patients in Iran. Immunol. Lett. 216, 70–78. 10.1016/j.imlet.2019.10.001 31589898

[B9] Shields B. E. Perelygina L. Samimi S. Haun P. Leung T. Abernathy E. (2021). Granulomatous dermatitis associated with rubella virus infection in an adult with immunodeficiency. JAMA Dermatol 157 (7), 842–847. 10.1001/jamadermatol.2021.1577 34037685 PMC8156178

[B10] Strubbe S. De Bruyne M. Pannicke U. Beyls E. Vandekerckhove B. Leclercq G. (2021). A novel non-coding variant in DCLRE1C results in deregulated splicing and induces SCID through the generation of a truncated ARTEMIS protein that fails to support V(D)J recombination and DNA damage repair. Front. Immunol. 12, 674226. 10.3389/fimmu.2021.674226 34220820 PMC8248492

[B11] Wanat K. A. Perelygina L. Chen M. H. Hao L. Abernathy E. Bender N. R. (2022). Association of persistent rubella virus with idiopathic skin granulomas in clinically immunocompetent adults. JAMA Dermatol 158 (6), 626–633. 10.1001/jamadermatol.2022.0828 35338705 PMC8957700

[B12] Xiao F. Lu Y. Wu B. Liu B. Li G. Zhang P. (2021). High-frequency exon deletion of DNA cross-link repair 1C accounting for severe combined immunodeficiency may Be missed by whole-exome sequencing. Front. Genet. 12, 677748. 10.3389/fgene.2021.677748 34421990 PMC8372405

