# Supplementary material for: Case report: Rubella virus-induced cutaneous granulomas in a girl with atypical SCID caused by DCLRE1C gene mutations
Source: Front Genet. 2023 Mar 16;14:1115027. doi: 10.3389/fgene.2023.1115027 (PMC10061095; doi:10.3389/fgene.2023.1115027)
Supplement: Supplementary file 1 [file DataSheet1.pdf]

# *Supplementary Material*

# Case Report: Rubella virus-induced cutaneous granulomas in a girl with atypical SCID caused by DCLRE1C gene mutations

**Sihan Deng [†1], Shijia Rao[†2], Alun R. Wang[3], Wei Shi[1,4]**

[1] Department of Dermatology, Xiangya Hospital, Central South University, Changsha, Hunan, PR China.

[2] Department of Dermatology, The Second Xiangya Hospital, Central South University, Hunan Key Laboratory of Medical Epigenomics, Changsha 410011, Hunan, PR China.

[3]Department of Pathology, Tulane University School of Medicine, New Orleans, LA, USA

[4] National Clinical Research Center for Geriatric Disorders, Xiangya Hospital, Central South University, Changsha, Hunan, China;

†These authors contributed equally to this work and share first authorship.

*** Correspondence:**
Wei Shi, MD, PhD

Department of Dermatology, Xiangya Hospital, Central South University, Changsha, Hunan, PR China.

E-mail: shiwei@csu.edu.cn

## STable 1：The laboratory results of this patient.

| The laboratory results of this patient. | | | | | | | | | | | | | | | | | | | | | | | |
|---|---|---|---|---|---|---|---|---|---|---|---|---|---|---|---|---|---|---|---|---|---|---|---|
| | | 2021-11-25 | | 2021-12-10 | | 2022-1-12 | | 2022-1-20 | | 2022-02-22 | | 2022-03-16 | | 2022-3-19 | | 2022-3-26 | | 2022-3-29 | | 2022-5-5 | | 2023-2-6 | |
| | | Result | Normal Range | Result | Normal Range | Result | Normal Range | Result | Normal Range | Result | Normal Range | Result | Normal Range | Result | Normal Range | Result | Normal Range | Result | Normal Range | Result | Normal Range | Result | Normal Range |
| | C3 (g/L) | 0.47 | 0.79-1.52 | 0.41 | 0.79-1.52 | - | - | - | - | - | - | 0.6 | 0.7-1.4 | - | - | - | - | - | - | - | - | 0.35 | 0.1-0.4 |
| | C4 (g/L) | 0.26 | 0.16-0.38 | 0.21 | 0.16-0.38 | - | - | - | - | - | - | 0.215 | 0.1-0.4 | - | - | - | - | - | - | - | - | 0.54 | 0.79-1.5 |
| | IgG (g/L) | 4.3 | 3.6-10.6 | 4.3 | 3.6-10.6 | - | - | 10.3 | 6-12 | - | - | 5.59 | 5.4-13.4 | - | - | - | - | - | - | - | - | 4.57 | 6.3-15.0 |
| | IgM (g/L) | 0.21 | 0.38-1.44 | 0.27 | 0.38-1.44 | - | - | 0.185 | 0.4-1.8 | - | - | 0.11 | 0.43-1.52 | - | - | - | - | - | - | - | - | 0.36 | 0.47-2.20 |
| | IgA (g/L) | 0.66 | 0.14-1.38 | ＜0.07 | 0.14-1.38 | - | - | <0.067 | 0.41-2.97 | - | - | 0.12 | 0.3-1.48 | - | - | - | - | - | - | - | - | ＜0.067 | 0.46-2.51 |
| | IgE (IU/mL) | ＜5.00 | ＜60 | ＜5.00 | ＜60 | - | - | ＜5.00 | ＜155 | - | - | 1 | 0-100 | - | - | - | - | - | - | - | - | | |
| Blood routine | WBC (×10^9/L ) | 5.9 | 4.3-11.3 | 6.49 | 4.3-11.3 | 3.7 | 4.6-11.9 | 3.65 | 4.6-11.9 | 7.92 | 4.9-12.7 | 2.52 | 3.5-9.5 | 3.17 | 3.5-9.5 | 2.63 | 3.5-9.5 | 2.84 | 3.5-9.5 | 5.53 | 4.4-11.9 | 4.3 | 4.6-11.3 |
| | Netrohphils (%) | 0.767 | 0.31-0.71 | 0.788 | 0.31-0.71 | 65.9 | 32-71 | 65 | 32-71 | 74.4 | 23-64 | 57.2 | 31-56 | 61.5 | 31-56 | 61.6 | 31-56 | 58.4 | 31-56 | 30.1 | 22-65 | 88.1 | 31-70 |
| | lymphocytes (×10^9/L ) | 0.649 | 1.5-4.6 | 0.941 | 1.5-4.6 | 0.87 | 1.7-4.7 | 0.9 | 1.7-4.7 | 0.96 | 1.8-6.3 | 0.49 | 1.1-3.2 | 0.58 | 1.1-3.2 | 0.53 | 1.1-3.2 | 0.67 | 1.1-3.2 | 2.94 | 1.8-6.3 | 0.4 | 1.5-4.6 |
| | monocytes (%) | 10.3 | 2-11 | 5.4 | 2-11 | 9.2 | 2-11 | 9.3 | 2-11 | 11.4 | 2-11 | 12.7 | 3-10 | 13.2 | 3-10 | 13.3 | 3-10 | 13 | 3-10 | 15 | 2-11 | 1.6 | 2-11 |
| | CD3+lymphocytes (%) | - | - | - | - | - | - | 34.93 | 50-84 | - | - | - | - | - | - | - | - | - | - | - | - | 70.37 | 58.7-75.0 |
| | CD3+CD4+lymphocytes (%) | - | - | - | - | - | - | 51.11 | 30.00-67.00 | - | - | - | - | - | - | - | - | - | - | - | - | 38.4 | 26.3-46.5 |
| | CD3+CD8+lymphocytes (%) | - | - | - | - | - | - | 41.19 | 23.00-50.00 | - | - | - | - | - | - | - | - | - | - | - | - | 26.63 | 16.0-29.5 |
| | CD3+CD8-lymphocytes (%) | - | - | - | - | - | - | 58.81 | 50.00-75.00 | - | - | - | - | - | - | - | - | - | - | - | - | - | - |
| | CD3+CD8-IL-4+lymphocytes (%) | - | - | - | - | - | - | 3.86 | 0.30-2.20 | - | - | - | - | - | - | - | - | - | - | - | - | - | - |
| | CD3+CD8-IFN-γlymphocytes+ (%) | - | - | - | - | - | - | 38.06 | 6.50-28.00 | - | - | - | - | - | - | - | - | - | - | - | - | - | - |
| | CD3+CD4+CD25+FOXP3+lymphocytes (%) | - | - | - | - | - | - | 3.25 | 4.10-9.40 | - | - | - | - | - | - | - | - | - | - | - | - | - | - |
| | CD3+CD8-IL-17A+lymphocytes (%) | - | - | - | - | - | - | 0.65 | 0.20-2.40 | - | - | - | - | - | - | - | - | - | - | - | - | - | - |
| | T helper cell (/uL) | - | - | - | - | - | - | - | - | - | - | - | - | 258 | 550-1440 | - | - | - | - | - | - | - | - |
| | T inhibited cell (/uL) | - | - | - | - | - | - | - | - | - | - | - | - | 185 | 320-1250 | - | - | - | - | - | - | - | - |
| | B lymphocyte (%) | - | - | - | - | - | - | - | - | - | - | - | - | 3.62 | 5-18 | - | - | - | - | - | - | - | - |
| | B lymphocyte (/uL) | - | - | - | - | - | - | - | - | - | - | - | - | 23 | 90-560 | - | - | - | - | - | - | - | - |
| | NK cell (/uL) | - | - | - | - | - | - | - | - | - | - | - | - | 128 | 150-1100 | - | - | - | - | - | - | - | - |
| | Total T lymphocyte (/uL) | - | - | - | - | - | - | - | - | - | - | - | - | 486 | 955-2860 | - | - | - | - | - | - | - | - |
| TBNK | CD3(%) | | | | | | | 66.6 | 55.0-82.0 | - | - | - | - | - | - | - | - | - | - | - | - | - | - |
| | CD8(%) | | | | | | | 22.0 | 25.0-57.0 | - | - | - | - | - | - | - | - | - | - | - | - | - | - |
| | CD4(%) | | | | | | | 37.9 | 14.0-34.0 | - | - | - | - | - | - | - | - | - | - | - | - | - | - |
| | CD4/8 | | | | | | | 1.72 | 1.1-2.0 | - | - | - | - | - | - | - | - | - | - | - | - | - | - |
| | TH/TS | | | | | | | - | - | - | - | - | - | - | - | - | - | - | - | - | - | - | - |
| | CD16+56(%) | | | | | | | 24.5 | 7.0-40.0 | - | - | - | - | - | - | - | - | - | - | - | - | - | - |
| | CD19(%) | | | | | | | 3.1 | 9-29 | - | - | - | - | - | - | - | - | - | - | - | - | - | - |
